# MRI Outcomes Achieved by Simple Flow Blockage Technique in Symptomatic Carotid Artery Stenosis Stenting

**DOI:** 10.3390/jpm12101564

**Published:** 2022-09-23

**Authors:** Jean-François Hak, Caroline Arquizan, Federico Cagnazzo, Mehdi Mahmoudi, Francois-Louis Collemiche, Gregory Gascou, Pierre-Henry Lefevre, Imad Derraz, Julien Labreuche, Isabelle Mourand, Nicolas Gaillard, Lucas Corti, Mahmoud Charif, Vincent Costalat, Cyril Dargazanli

**Affiliations:** 1 Neuroradiology Department, CHRU Gui de Chauliac, Montpellier University Medical Center, 34295 Montpellier, France; 2 LIIE, Aix Marseille University, 13005 Marseille, France; 3 CERIMED, Aix Marseille University, 13005 Marseille, France; 4 Neurology Department, CHRU Gui de Chauliac, Montpellier University Medical Center, 34295 Montpellier, France; 5 Biostatistics Department, Centre Hospitalier Universitaire Lille, 59000 Lille, France

**Keywords:** carotid, endovascular, stents, magnetic resonance imaging, diffusion-weighted-imaging

## Abstract

In this study, we aimed to determine the frequency and clinical impact of new ischemic lesions detected with diffusion-weighted-imaging-MRI (DWI-MRI) as well as the clinical outcomes after carotid artery stenting (CAS) using the simple flow blockage technique (SFB). This is a retrospective study with data extraction from a monocentric prospective clinical registry (from 2017 to 2019) of consecutive patients admitted for symptomatic cervical ICA stenosis or web. Herein, patients benefited from DWI-MRI before and within 48 h of CAS for symptomatic ICA stenosis or web. The primary endpoint was the frequency of new DWI-MRI ischemic lesions and the secondary (composite) endpoint was the rate of mortality, symptomatic stroke or acute coronary syndrome within 30 days of the procedure. All of the 82 CAS procedures were successfully performed. Among the 33 patients (40.2%) with new DWI-MRI ischemic lesions, 30 patients were asymptomatic (90.9%). Irregular carotid plaque surface with (*n* = 13, 44.8%) or without ulceration (*n* = 12, 60.0%) was associated with higher rates of new DWI-MRI lesions by comparison to patients with a regular plaque (*n* = 7, 25%) (*p* = 0.048) using the univariate analysis. Less than half of this CAS cohort using the SFB technique had new ischemic lesions detected with DWI-MRI. Among these patients, more than 90% were asymptomatic. Irregularity of the plaque seems to increase the risk of peri-procedural DWI-MRI lesions.

## 1. Introduction

Symptomatic carotid stenosis, defined as stenosis of the internal carotid artery (ICA) leading to recent symptoms of amaurosis fugax, transient ischemic attacks or acute ischemic stroke (AIS) ipsilateral to the lesion [1], carries a high risk of early recurrence and stroke and carotid revascularization is recommended [2]. Randomized controlled trials (EVA-3S, SPACE, and ICSS) comparing carotid endarterectomy (CEA) and carotid artery stenting (CAS) have shown a higher periprocedural risk of stroke with CAS than with CEA [3,4,5]. However, risks of stroke or death in patients younger than 70 years were similar in both treatment groups [6]. Therefore, CAS has been developed during the past two decades to treat symptomatic carotid and is often chosen for anatomic reasons (contralateral occlusion), for patients with high surgical risk (radiation injury, history of prior neck dissection, presence of tracheostomy) or with severe medical comorbidities (high risk for open surgery).

Carotid web (CW) leads to ischemic stroke secondary to blood flow stasis and subsequent embolization. In addition, optimal management strategies for secondary stroke prevention remain unclear. Due to the high stroke recurrence rate in medically managed symptomatic CW patients, carotid revascularization is currently performed, with no periprocedural complications or recurrent strokes in carotid revascularization management with CAS or CEA [7].

Moreover, the recent numerous randomized controlled trials (RCT) established mechanical thrombectomy as the gold standard for the treatment of AIS with large vessel occlusion, with the possibility of emergency carotid stenting in tandem occlusion [8]. This post-thrombectomy era led to an increased number of endovascular treatments of AIS during the past few years. Nevertheless, the current key point is the safety of CAS. During these last years, the technical and medical experience of the operators, as well as new innovative endovascular techniques, devices, and a better management of the antiplatelet therapies could reduce this procedural risk. 

Indeed, the embolic protection technique during CAS as proximal balloon occlusion (PBA), and distal filter protection (DFP) can provide similar levels of protection from periprocedural stroke and 30-day mortality [9]. In the dynamic of developing the safest CAS procedure, an innovative approach (simple flow blockage technique [SFB]) was reported as a combination of proximal balloon occlusion and flow blockage, which is inspired from a mechanical thrombectomy technique [10].

Diffusion-weighted-imaging-MRI (DWI-MRI) remains the gold standard to evaluate acute stroke lesions even without any clinical significance. The objective of this study is to report the frequency of new ischemic lesions using DWI-MRI as well as their impact on clinical outcomes after consecutive carotid artery stenting (CAS) is performed with SFB in consecutive patients with AIS (without large vessel occlusion) and symptomatic carotid artery stenosis or carotid web.

## 2. Materials and Methods

### 2.1. Study Design

We carried out a retrospective study with data extraction from our prospective clinical registry of consecutive patients admitted for symptomatic ICA atherosclerotic stenosis or carotid web to our Comprehensive Stroke Center (CSC) from January 2017 to September 2019. 

This study (reference study RECHMPL 18 0236, No. ID-RCB: 2018-A02651-54) was approved by a local ethics committee (Comité de Protection des Personnes (CPP) SUD-MEDITERRANEE III, UFR MEDECINE 186, chemin du Carreau de Lanes CS 83021 30908 NIMES Cedex 2), and registered on clinicaltrials.gov (NCT04421326).

In the context of carotid stenting using the simple flow blockage technique, we previously reported data from January 2015 to 2018 in 75 patients, focusing on the angiographic pattern [10]. In this previous study, only 40 patients (53.3% of the cohort) benefited from a MRI follow-up [10]. 

For this current study, focusing on a routine MRI protocol follow-up implemented in our center since January 2017 for CAS, we included 15 patients who were already analyzed in the previous study (from year 2017).

### 2.2. Inclusion Criteria

Consecutive patients were included if they fulfilled the following criteria: (I) AIS (hemispheric or retinal TIA), cerebral infarct in the anterior circulation without large vessel intracranial occlusion <3 months before admission; (II) pre-procedural (<1 month) brain DWI-MRI (diffusion-weighted-imaging-MRI) showing cytotoxic lesions of AIS and/or perfusion-MRI showing brain hypoperfusion; (III) ipsilateral atheromatous internal carotid artery stenosis >50% or carotid web. The diagnosis of carotid stenosis or web was performed on noninvasive imaging (CT and/or MRI); (IV) CAS for ICA atheromatous stenosis ≤50% was retained in the case of plaque instability as demonstrated by an intraplaque hemorrhage on dedicated black-blood MRI; (V) post-procedural (<48 h) brain DWI-MRI. The decision of endovascular treatment was retained after a multidisciplinary team meeting. 

The exclusion criteria were: (1) Patients with intracranial large vessel occlusion; (2) patients with pre-stroke dependency defined as a modified Rankin score > 2; (3) patients with severe or fatal co-morbidities or life expectancy under 6 months; (4) patients treated endovascularly using a DFP device; and (5) patients with carotid artery stenosis at the acute phase of ischemic stroke caused by tandem occlusion.

### 2.3. Patient Management

#### 2.3.1. Medical Management

All patients were hospitalized in the CSC for maintenance of hemodynamic function (objective of systolic blood pressure > 120 mmHg), and close monitoring of any neurological worsening. Anticoagulants and/or aspirin was left to the discretion of the stroke neurologist. 

Clopidogrel (75 mg daily) was given within the days before CAS, with an assessment of platelet inhibition (PRU, Platelet Reactive Units) (VerifyNow, Instrumentation Laboratory, San Diego, CA, USA) before the procedure. 

In cases of clopidogrel resistance (230–240 P2Y12 reaction units PRU by the VerifyNow P2Y12 assay or platelet inhibition rate < 20%) [10], prasugrel (20 mg) was given the day before the intervention and platelet inhibition was tested again before the procedure. 

All patients had subsequently 3 months of dual antiplatelet medication (aspiring 75 mg), then lifelong aspirin. 

In cases of emergency stenting for hemodynamic symptoms or early recurrence, a loading dose of platelet inhibitor (300 mg of clopidogrel or 20 mg of prasugrel) was administered and platelet inhibition was then assessed.

All procedures were performed via a femoral artery approach, and 150 IU/kg heparin was administered intravenously after the placement of the 9-French balloon guiding catheter (Concentric medical, Mountain View, CA, USA) into the targeted common carotid artery. The balloon guiding catheter was prepared according to the instructions provided by the manufacturer, using a combination of contrast agent with saline (50% by volume) to prepare balloon inflation media.

Atropine (0.01–0.02 mg/kg) was administered intravenously at the time of angioplasty, which was performed using a 4.0–7.0 mm balloon (Ultra-soft SV monorail balloon, Boston Scientific, Marlborough, MA, USA).

#### 2.3.2. Simple Flow Blockage (SFB) Technique

All procedures were performed under conscious sedation by experienced interventional neuroradiologists. A baseline angiographic run was performed after the inflation of the balloon guiding catheter in the common carotid artery, and was allowed to determine the different angiographic patterns [10].

In all cases, flushing of the guiding catheter was temporarily interrupted during the critical steps (crossing of culprit lesions, stent deployment, angioplasty) to avoid antegrade embolization. 

After a dangerous maneuver (crossing of culprit lesions, stent deployment, angioplasty), two steps were respected: (1) Pump aspiration (Penumbra, Alameda, CA, USA) set on the recommended vacuum pressure of −25.5 inches Hg (−86.4 kPa) via a rotating hemostatic valve (RHV) for approximately 10 s before balloon deflation, to avoid potential embolization from the stagnating column of blood distal to the balloon or from the guiding catheter itself; and (2) after balloon deflation, the RHV is opened for a few seconds and closed progressively during activation of the flushing line, to assure that the RHV is clean (aspiration is performed through it in prevention of the accumulated debris inside the RHV). 

A DFP device was not used during any procedures in this study. The type of stent and the use of pre-dilation and post-dilation by balloon angioplasty were performed according to neurointerventionalists’ discretion. 

Balloon angioplasty was not performed for web cases.

#### 2.3.3. Follow-Up and Outcome

All patients were hospitalized in the CSC for at least 24 h following the intervention, with strict blood pressure monitoring and management (target level of <140/90 mmHg) and close monitoring of any neurological worsening. 

Patients with preprocedural severe stenosis (>90%), severe contralateral stenosis or occlusion, or with periprocedural arterial hypertension were considered at high risk of reperfusion syndrome, leading to a stricter blood pressure monitoring and management with a target level of <120/80 mmHg. Patients were treated after the procedure with clopidogrel (75 mg daily) or with prasugrel (10 mg daily) in the case of clopidogrel resistance (in the absence of contraindication). This treatment was delayed by 6 to 24 h for patients treated with intravenous thrombolysis.

A post-procedural (<48 h) follow-up cerebral MRI was systematically performed after stenting.

Patients were discharged 48–72 h post-treatment, after a comprehensive neurological evaluation. 

The primary endpoint was the retrospective assessment of new ischemic lesions detected by DWI-MRI by two neurointerventionalists (JFH and CD) blinded to the clinical outcomes and the procedure success (defined as the ability to successfully implant a carotid stent with <30% residual stenosis). The secondary (composite) endpoint was the rate of mortality, symptomatic stroke or acute coronary syndrome within 30 days of the procedure.

The new symptomatic stroke was defined as a neurological deficit, as explained by a new ischemic lesion on DWI.

### 2.4. MRI Evaluation

MRI scans were performed using a Siemens Avanto 1.5 T.

On the 1.5 T scanner, the sequence parameters were: TR = 24 ms, TE = 6.00 ms, flip angle = 90.3 directions of measurement, 16 cm FOV, 131 × 131 matrix, and 5 mm section thickness. Twelve-channel head coils were used. Foci of diffusion were measured in the longest axial axis. DWI b-value was b = 1000 for all studies. All patients were scanned with the same protocol before CAS and within the 48-h following the CAS procedure.

### 2.5. Data Collection

Patient’s characteristics, imaging data, treatment, and follow-up were prospectively collected at the CSC: (a) Clinical data: Age, gender, medication, and vascular risk factors (hypertension, hypercholesterolemia, diabetes, current smoking); (b) imaging data: Type of initial arterial imaging (MRA, CTA; type of carotid stenosis: Degree of stenosis, web, plaque surface characteristics (ulcerated, irregular)). 

Pre- and post-procedural (<48 h) DWI-MRI (diffusion-weighted-MRI) were performed for all patients.

Data supplemental to the main text regarding data collection, patients’ management (medical management and SFB technique, follow-up, and outcome) can be found in Appendix A.

### 2.6. Statistical Analysis

Categorical variables were expressed as frequencies and percentages and continuous as mean (standard deviation) or median (interquartile range, IQR) in the case of non-normal distribution. Normality of distribution was assessed graphically and using the Shapiro-Wilk test. Rates of outcomes were estimated by calculating exact binomial 95% confidence intervals (CIs). Bivariate comparisons in main ICA lesions and treatment characteristics between patients with and without new cerebral lesions at DWI-MRI were conducted using the Chi-Square test (or Fisher’s exact test when the expected cell frequency was <5) for categorical variables, and Mann-Whitney U test for continuous variables as appropriate. Statistical testing was conducted at the two-tailed α-level of 0.05. Data were analyzed using the SAS software package, release 9.4 (SAS Institute, Cary, NC, USA).

## 3. Results

### 3.1. Patient Characteristics and Angiographic Patterns

**Figure 1:**
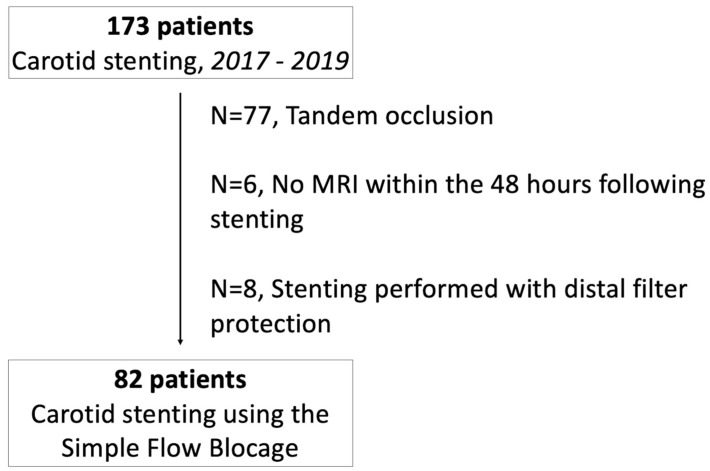
Flow-Chart.

From January 2017 to September 2019, 82 patients with symptomatic carotid artery atherosclerotic stenosis (*n* = 78) or symptomatic web (*n* = 4) were treated by CAS using the SFB technique in our CSC and benefited from a DWI-MRI before and within 48 h of the stenting procedure (see flowchart; Figure 1). Patient and treatment characteristics are reported in Table 1. Overall, the median age was 68 years (IQR, 59 to 75), 37 patients (45.1%) were men. Baseline median NIHSS was 5.6 (IQR, 1 to 18). CAS was performed after a median time from symptom of 7 days (IQR, 3 to 30 days). Among the 78 patients with atherosclerotic stenosis (95.1%), the median degree of carotid stenosis was 75% (IQR, 70 to 90), ulcerations were present in 63.7% of cases. 

No resistance to clopidogrel was observed in this cohort.

Complete stagnation of the contrast column in the ICA (angiographic pattern 1) was observed in 28 patients (34.1%), retrograde wash-out of the internal carotid artery from the intracranial circulation toward the external carotid artery (pattern 2) in 27 patients (32.9%), and antegrade wash-out of contrast toward the intracranial circulation, via the external carotid artery or from the common carotid artery (pattern 3) was observed in the remaining 27 cases (32.9%). CAS was performed using Casper (Carotid Artery Stent designed to Prevent Embolic Release, MicroVention Inc., Tustin, CA, USA) (*n* = 18), Xact (Abbott Vascular, Abbott Park, IL, USA) (*n* = 53) or Precise (Cordis, Warren, NJ, USA) (*n* = 17) stents with a median procedure time of 35 min (IQR, 24 to 45 min). For 11 patients, two or three stents were used (see footnote of Table 1). All the CAS procedures were successfully achieved.

### 3.2. Clinical and DWI Outcomes

Within the 48 h after CAS, a total of 177 new cerebral lesions (134 punctiform lesions, 32 lesions ≤ 10 mm and 11 lesions > 10 mm) were detected by DWI-MRI in 33 patients (40.2%, 95%CI: 29.5 to 51.7%, Table 2). Thirty-one patients (37.8%) had at least one punctiform lesion, thirty-one (37.8%) had at least one ipsilateral lesion, and seven (8.5%) had at least one lesion > 10 mm. 

**Figure A1:**
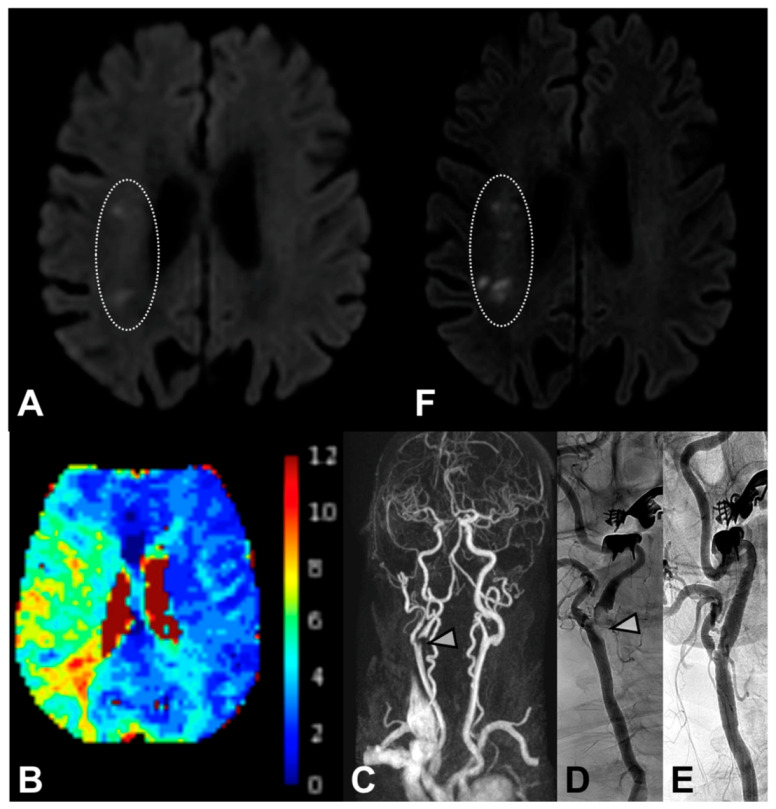
Example of new punctiform ischemic DWI-MRI lesions for a patient with fluctuant left-sided hemiparesis. Initial MRI (5 h from onset) did not find any intracranial large vessel occlusion, but DWI-MRI depicted punctiform ischemic lesions in the right anterior junctional area. (**A**) Perfusion MRI showed a large right sylvian hypoperfusion (TMax > 6 s). (**B**) MR-angiography of supra-aortic arteries objectified a severe proximal ICA stenosis (**C**; white arrow) confirmed by DSA performed (130 h after onset) before (**D**; white arrow) a successful carotid artery stenting (**E**). DWI-MRI performed 24 h after CAS objectified new silent punctiform DWI-MRI lesions in the same right anterior junctional area (**F**).

Eight patients (9.8%) had contralateral lesions. Among these 33 patients, the baseline median NIHSS was 7.2 (IQR, 1 to 18). Thirty patients (36.6%) had new ischemic DWI-MRI lesions without new neurological symptoms or modification of the NIHSS during periprocedural follow-up. An example of the new punctiform ischemic DWI-MRI lesions is provided in Appendix B Figure A1.

**Figure A2:**
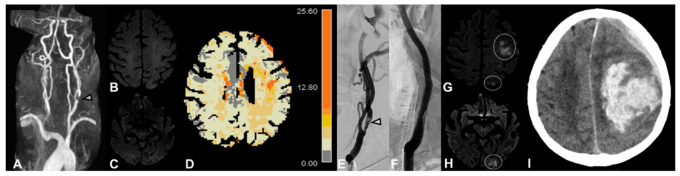
This patient presented right hemiparesis with a symptomatic 75% left ICA stenosis confirmed with angio-MR (**A**) without any ischemic brain lesions on the DWI-MRI performed 8 h from symptom onset (**B**,**C**), but perfusion MRI showed left sylvian hypoperfusion (TMax > 6 s) (**D**). Carotid artery stenting performed 1 day after symptom onset confirmed the ICA stenosis (**E**) and the stenting was successfully performed (**F**). However, the patient presented pre-procedural hypertension involving strict blood pressure monitoring after the CAS with a target level of <120/80 mmHg. The DWI-MRI performed 25 h from the CAS revealed symptomatic new cerebral lesions (punctiform and >10 mm) in the ipsilateral side (**G**,**H**) without large intracranial vessel occlusion. Dual antiplatelet medication was continued. However, the 30-h CT performed for a neurologic deterioration revealed an intraparenchymal hemorrhage (**I**) corresponding to a reperfusion syndrome.

Three patients had symptomatic ischemic strokes with NIHSS degradation in two patients (respectively from NIHSS 6 and 7 to NIHSS 10 to 9) and death from the reperfusion syndrome in one patient. The latter was known with history of hypertension and hypercholesterolemia, and presented with a symptomatic 75% left ICA stenosis. Baseline NIHSS was 18 and DWI-MRI showed no initial ischemic lesions and no intracranial large vessel occlusion. The CAS procedure was successfully performed 1 day from symptoms onset, but the patient presented pre-procedural hypertension leading to strict blood pressure monitoring after the CAS with a target level of <120/80 mmHg. The DWI-MRI performed 25 h from the CAS revealed symptomatic new cerebral lesions (punctiform and >10 mm) both in the ipsilateral and contralateral side without large intracranial vessel occlusion. Dual antiplatelet medication was continued. A neurologic deterioration occurred, and the 30-h CT revealed an intraparenchymal hemorrhage corresponding to a reperfusion syndrome. The patient died 34 h after the CAS (see Appendix B Figure A2). 

No acute coronary syndrome was observed. Overall, the 30-day composite endpoint occurred in three patients (3.7%).

Association of ICA lesion and treatment characteristics with the presence of ≥1 new DWI-MRI lesions are reported in Table 3. Only carotid plaque surface characteristics were significantly associated with the presence of ≥1 new cerebral lesions; patients with irregular plaque with (*n* = 13, 44.8%) or without ulceration (*n* = 12, 60.0%) had a higher rate of new cerebral lesions by comparison to patients with regular plaque (*n* = 7, 25%) (*p* = 0.048).

## 4. Discussion

In this large cohort of 82 consecutive patients with symptomatic severe carotid stenosis or carotid web treated with a standardized procedure of stenting using the SFB technique, less than half of this CAS cohort had new ischemic lesions detected with DWI-MRI (*n* = 33; 40.2%), asymptomatic in 30 patients (90.9%). Three (3.7%) patients had a newly onset symptomatic ischemic stroke and one (1.2%) had a transient focal neurological deficit. Among the patients with newly onset symptomatic ischemic stroke, one patient (1.2%) experienced reperfusion syndrome.

Both CEA and CAS are known to have increased periprocedural complications in symptomatic patients. However, the risk-benefit balance tilts in favor of treatment, both with CAE [1] and CAS [11].

In a meta-analysis of three RCT (EVA-3S, SPACE, and ICSS), any stroke or death occurred significantly more often in the CAS group compared to the CEA group. Nevertheless, an analysis of individual patient data showed that risk with CAS was twice the risk of CEA in patients greater than age 70 but was the same under 70 [6]. In the same line, the risk for stroke, acute coronary syndrome or death with CAS significantly increased with age according to the CREST investigators [12].

The CREST [13,14] demonstrated equivalent composite outcomes (stroke, myocardial infarction, death) between CEA and CAS. However, the subgroup analysis suggested an increased risk of stroke with CAS. Similarly, a recent meta-analysis (Carotid Artery Stenting Versus Endarterectomy for Stroke Prevention: A Meta-Analysis of Clinical Trials) confirmed these findings [15]. 

However, over the past two decades, embolic protection devices emerged for CAS and allowed progressive improvements in terms of stroke and mortality rates [16].

More recently, and in contrast to the findings of CREST, Cole et al. suggested that 378,354 patients undergoing CEA had a higher rate of perioperative stroke than 57,273 patients undergoing CAS, primarily among symptomatic patients (8.1% versus 5.6%; odds ratio, 1.47 [CI: 1.29–1.68]; *p* < 0.001) [17]. 

Current guidelines advise proceeding with CEA for symptomatic carotid only if the surgeon’s rate for perioperative stroke or death is <6% [2,18]. In our cohort, perioperative stroke or death occurred for 3.7%, suggesting that progress of CAS, especially using the SFB technique, led to a very attractive approach for symptomatic carotid stenosis.

### 4.1. DWI Lesions

Clinical series [19] and systematic review [20] showed that patients treated by CAS have about three times more new ischemic lesions on DWI-MRI compared to the CAE group, respectively 37% and 50% in the CAS group, and 10% and 17% in the CAE group (*p* < 0.01). 

However, the main weakness of these results is the heterogeneity in the use of the protective device, as well as differences in the used material (balloon, DFP).

Interestingly, in the ICSS-MRI sub-study, Gensicke et al. found that there was no significant difference in total lesion volume per patient between the CAS and CEA groups in the entire study population (*p* = 0.18). Among these patients with silent ischemic DWI-MRI lesions after treatment (CAS, *n* = 62 patients; CEA, *n* = 18), volumes of separate lesions were significantly smaller in the CAS group (median volume, 0.02 mL) than the CEA group (0.08 mL; *p* < 0.0001) [21].

In addition, it is known that a routine diagnostic angiography induces silent ischemic lesions. Bendszus et al. showed that 44% of patients with vasculopathy who benefited from a routine diagnostic angiography had silent embolisms compared to a group without vascular risk factors (13%) [22]. Accordingly, the 40% rate of patients experiencing DWI-MRI lesions after CAS in our cohort is comparable to what was reported in the literature.

### 4.2. Irregular/Ulcerated Stenosis and Degree of Stenosis

Our study suggests that plaque surface characteristics were significantly associated with the presence of ≥1 new DWI cerebral lesions. Indeed, patients with irregular plaque with (*n* = 13, 44.8%) or without ulceration (*n* = 12, 60.0%) had a higher rate of new cerebral lesions in comparison to patients with regular plaque (*n* = 7, 25%) (Table 3). 

This is in accordance with other studies suggesting that the irregular plaque surface was associated with increased incidence of carotid stenting-associated ischemic lesions [23,24].

Exploratory analyses from EVA-3S [3,25], SPACE [26], and ICSS [27] cohorts did not show any significant association between the degree of stenosis and stroke after CAS or CAE with a 70% stenosis [26,27] or a 90% stenosis cut-off [3,25]. Interestingly, the ICSS cohort [27] evaluated fatal or disabling strokes of 3.9% and 6.7% after CAS and 9.6% and 6.2% after CAE, respectively, for an ipsilateral stenosis of 50–69% and 70–99% (*p* = 0.155).

According to our results using SFB and to the literature, the most important point associated with peri-procedural stroke in patients with stenosis > 50% may be the irregularity and not the severity of the stenosis after CAS.

### 4.3. Timing of Angioplasty

It is interesting to note that the delay from AIS onset and treatment with CAS was not associated with an increased risk of peri-procedural DWI-MRI lesions. Current guidelines are to pursue early revascularization within 14 days of AIS [2]. Due to the increasing risk of recurrence within the first 2 weeks after the first ischemic event of a symptomatic carotid stenosis, Liu et al. showed improved functional outcomes without increasing the rate of new AIS, myocardial infarction or death in the early CAS group (<1 week) compared to the delayed CAS at 1 month [28]. On the contrary, Song et al. evaluated CAS for 206 patients with moderate-to-severe stenosis and found a significantly higher rate of ipsilateral stroke or death (at 30 days) of 12.8% in the early CAS group (within 14 days) compared to only 0.8% in the delayed CAS group (mean timing of CAS was 52.6 ± 36.94 days) [29]. However, this finding did not extend beyond the 30-day follow-up period (31 days to 1 year), wherein there was no significant difference between groups. A post-hoc analysis with the CREST population did not find any relationship between timing and significant adverse events in the 583 patients in the CAS group [30], and this was in agreement to what we found in our study (Table 3).

Transcarotid artery revascularization (TCAR) with flow reversal was developed to mitigate the maneuvers at highest risk for causing stroke during transfemoral CAS.

Comparing TCAR to CEA across different age groups showed no significant differences in outcomes [31,32].

In comparison to transfemoral CAS for patients ≥ 80 years, TCAR was associated with a reduction in stroke risk, reduction in risk of stroke/death, and reduction in the risk of stroke/death/myocardial infarction of respectively 72%, 65%, and 76% [31,32]. However, these results must be set against the advances in the experience of interventional practitioners and their equipment, in particular the famous balloon-guiding-catheters. In addition, aspiration is now possible with mechanical thrombectomy devices and confirms the need to repeat randomized studies.

### 4.4. Study Limitations

There are several limitations in the current study. The number of patients in our analysis was relatively small and there was no CAE group for comparison. 

Moreover, this study only focused on patients who benefited from a DWI-MRI before and within the 48-h following a CAS procedure.

However, this current CAS cohort only included symptomatic patients with symptomatic carotid stenosis or web which carries higher risk than asymptomatic carotid, with a known tendency for more silent ischemic lesions after CAS, suggesting that patients with symptomatic carotid had an increased micro-embolic risk during the CAS procedure [24].

## 5. Conclusions

In this study, less than half of the CAS cohort using the SFB technique had new ischemic lesions detected with DWI-MRI. Among these patients, more than 90% were asymptomatic. As a result, irregularity of the plaque seems to increase the risk of peri-procedural DWI-MRI lesions.

## Figures and Tables

**Table 1:** Patient and treatment characteristics of the 82 included patients with symptomatic carotid stenosis >50% or symptomatic web treated by CAS using SFB.

Characteristics	*n*	Values
**Demographics**		
Age (years), median (IQR)	82	68 (59 to 75)
Men	82	37 (45.1)
**Medical history**		
Hypertension	82	67 (81.7)
Diabetes mellitus	82	33 (40.2)
Dyslipidemia	82	47 (57.3)
Current smoking	82	45 (54.9)
Long-term antithrombotic use	82	26 (31.7)
**Lesion characteristics**		
Location of lesion		
Right ICA	82	47 (57.3)
Left ICA		34 (41.5)
Bilateral ICA		1 (1.2)
Degree of carotid stenosis, %, median (IQR)	78	75 (70 to 90)
0 to 49 (including web)		6 (7.7)
50 to 69		13 (16.7)
70 to 99		59 (75.6)
Etiology		
Atherosclerosis	82	78 (95.1)
Web		4 (4.9)
Carotid plaque surface characteristics		
Regular	77	28 (36.4)
Irregular without ulceration		20 (26.0)
Irregular with ulceration		29 (37.7)
Angiographic Pattern		
Pattern 1	82	28 (34.3)
Pattern 2		27 (32.9)
Pattern 3		27 (32.9)
**Treatment characteristics**		
Onset to endovascular procedure, days, median (IQR)	82	7 (3 to 30)
Duration of endovascular procedure, minutes, median (IQR)	82	35 (25 to 45)
Type of stent ^1^		
Casper (Microvention, Aliso Viejo, CA, USA)	95	21 (22.1)
Xact (Abbott Vascular, Santa Clara, CA, USA)		56 (59.6)
Precise (Cordis, Milpitas, CA, USA)		18 (18.9)

^1^: Two patients with three stents (one with three Precise and one with three Xact), and nine patients with two stents (six with Xact and Precise, two with Casper and Xact, and one with Precise). Pattern 1: No anterograde flow; Pattern 2: Wash-out of the internal carotid artery by the external carotid artery; Pattern 3: Retrograde wash-out of the internal carotid artery by the intracranial circulation. Abbreviations: CAS: Carotid artery stenting; ICA: Internal carotid artery; IQR: Interquartile range; SFB: Simple flow blockage.

**Table 2:** DWI-MRI new lesions and clinical outcomes in 82 patients with symptomatic carotid stenosis >50% or symptomatic web treated by CAS using SFB.

Outcomes	*n* (%)	95%CI
**DWI-MRI new lesions**		
Patients with ≥ 1 lesions	33 (40.2)	29.5 to 51.7
*Single*	*11 (13.4)*	
*Multiple*	*22 (26.8)*	
Patients with punctiform lesions	31 (37.8)	27.3 to 49.2
Patients with ipsilateral lesions	31 (37.8)	27.3 to 49.2
Patients with contralateral lesions	8 (9.8)	4.3 to 18.3
Patients with lesions > 10 mm	7 (8.5)	3.5 to 16.8
**Clinical outcomes**		
Composite endpoint at 30 days of intervention	3 (3.7)	1.3 to 12.0
*Death*	*1 (1.2)*	
*Acute coronary syndrome*	*0 (0.0)*	
*Symptomatic ischemic stroke*	*3 (3.7)*	
≥1 Bleeding event	1 (1.2)	
≥1 procedural complications	3 (3.7)	1.3 to 12.0
*Cerebral emboli*	*3 (3.7)*	
*Groin hematoma*	*0 (0.0)*	
*Dissection*	*0 (0.0)*	

**Table 3:** Comparisons of ICA lesion and treatment characteristics between patients with and without new cerebral lesions detected by DWI-MRI.

	No DWI-MRI Lesions (*n* = 49)	≥1 DWI-MRI Lesions (*n* = 33)	*p*-Value
**ICA lesion characteristics**			
Stenosis degree, %			
0 to 49	2 (4.4)	4 (12.1)	0.41
50 to 69	7 (15.6)	6 (18.2)	
70 to 99	36 (80.0)	23 (69.7)	
Carotid plaque surface			
Regular	21 (46.7)	7 (21.9)	0.048
Irregular without ulceration	8 (17.8)	12 (37.5)	
Irregular with ulceration	16 (35.6)	13 (40.6)	
Angiographic Pattern			
Pattern 1	18 (40.0)	8 (25.8)	0.30
Pattern 2	15 (33.3)	10 (32.3)	
Pattern 3	12 (26.7)	13 (41.9)	
**Treatment characteristics**			
Number of stents			
One	43 (87.8)	28 (84.9)	0.75
Two or three	6 (12.2)	5 (15.2)	
Duration of endovascular procedure, minutes, median (IQR)	38 (30 to 45)	29 (24 to 45)	0.17
Delay from symptoms onset to endovascular procedure, days, median (IQR)	11 (3 to 30)	5 (0.8 to 31)	0.17

Values are % (*n*) unless otherwise as indicated. Abbreviations: DWI-MRI: Diffusion-weighted-imaging-magnetic resonance imaging; ICA: Internal carotid artery; IQR: Interquartile range.

## Data Availability

The data presented in this study are available on request from the corresponding author.

## Appendix A

### Appendix A.1. Data Collection

On the 1.5 T scanner, the sequence parameters were: TR = 24 ms, TE = 6.00 ms, flip angle = 90.3 directions of measurement, 16 cm FOV, 131 × 131 matrix, and 5 mm section thickness. Twelve-channel head coils were used. Foci of diffusion were measured in the longest axial axis. DWI b-value was b = 1000 for all studies.

### Appendix A.2. Patients’ Management

#### Appendix A.2.1. Medical Management and Simple Flow Blockage Technique (SFB)

Clopidogrel (75 mg daily) was given within the days before CAS, with assessment of platelet inhibition (PRU, Platelet Reactive Units) (VerifyNow, Instrumentation Laboratory, San Diego, CA, USA) before the procedure.

In cases of clopidogrel resistance (230–240 P2Y12 reaction units PRU by the VerifyNow P2Y12 assay or platelet inhibition rate <20%), prasugrel (20 mg) was given the day before the intervention and platelet inhibition was tested again before the procedure.

All patients had subsequently 3 months of dual antiplatelet medication (aspiring 75 mg), then lifelong aspirin.

In cases of emergency stenting for hemodynamic symptoms or early recurrence, a loading dose of platelet inhibitor (300 mg of clopidogrel or 20 mg of prasugrel) was administered and platelet inhibition was then assessed.

All procedures were performed via a femoral artery approach, and 150 IU/kg heparin was administered intravenously after the placement of the 9-French balloon guiding catheter (Concentric medical, Mountain View, CA, USA) into the targeted common carotid artery. The balloon guiding catheter was prepared according to the instructions provided by the manufacturer, using a combination of contrast agent with saline (50% by volume) to prepare balloon inflation media.

Atropine (0.01–0.02 mg/kg) was administered intravenously at the time of angioplasty, which was performed using a 4.0–7.0 mm balloon (Ultra-soft SV monorail balloon, Boston Scientific, Marlborough, MA, USA).

All procedures were performed under conscious sedation by experienced interventional neuroradiologists. A baseline angiographic run was performed after the inflation of the balloon guiding catheter in the common carotid artery, and allowed to determine the different angiographic patterns.

In all cases, flushing of the guiding catheter was temporarily interrupted during the critical steps (crossing of culprit lesions, stent deployment, angioplasty) to avoid antegrade embolization.

After a dangerous maneuver (crossing of culprit lesions, stent deployment, angioplasty), two steps were respected: (1) Pump aspiration (Penumbra, Alameda, CA, USA) set on the recommended vacuum pressure of −25.5 inches Hg (−86.4 kPa) via a rotating hemostatic valve (RHV) for approximately 10 s before balloon deflation, to avoid potential embolization from the stagnating column of blood distal to the balloon or from the guiding catheter itself; and (2) after balloon deflation, the RHV is opened for a few seconds and closed progressively during activation of the flushing line, to assure that the RHV is clean (aspiration is performed through it in prevention of accumulated debris inside the RHV).

#### Appendix A.2.2. Follow-Up and Outcome

Patients with preprocedural severe stenosis (>90%), severe contralateral stenosis or occlusion, or with periprocedural arterial hypertension were considered at high risk of reperfusion syndrome, leading to a stricter blood pressure monitoring and management with a target level of <120/80 mmHg. Patients were treated after the procedure with clopidogrel (75 mg daily) or prasugrel (10 mg daily) in the case of clopidogrel resistance (in the absence of contraindication). This treatment was delayed by 6 to 24 h for patients treated with intravenous thrombolysis.
